# Stereoselectivity of supported alkene metathesis catalysts: a goal and a tool to characterize active sites

**DOI:** 10.3762/bjoc.7.3

**Published:** 2011-01-05

**Authors:** Christophe Copéret

**Affiliations:** 1 Université de Lyon, Institut de Chimie de Lyon, Laboratoire C2P2 UMR 5265 (CNRS – CPE – UCBL) CPE Lyon, 43 Bd du 11 Novembre 1918, F-69616 Villeurbanne Cedex, France; 2 Department of Chemistry, ETH Zürich, Wolfgang-Pauli-Str. 10, CH-8093 Zürich, Switzerland

**Keywords:** active sites, metathesis, stereoselectivity, supported catalysts

## Abstract

Stereoselectivity in alkene metathesis is a challenge and can be used as a tool to study active sites under working conditions. This review describes the stereochemical relevance and problems in alkene metathesis (kinetic vs. thermodynamic issues), the use of (*E*/*Z*) ratio at low conversions as a tool to characterize active sites of heterogeneous catalysts and finally to propose strategies to improve catalysts based on the current state of the art.

## Introduction

Achieving high selectivity and, in particular, stereoselectivity are still important goals in organic synthesis, and several catalytic reactions such as alkene oxidation [1–2], hydrogenation [3], polymerisation [4], especially when using homogeneous catalysts, have reached a very high level of chemo-, diastereo- and enantioselectivy. In contrast, while alkene metathesis has been regarded as a powerful tool to introduce new C–C bonds into an organic skeleton and to generate alkenes [5–7], controlling the stereochemical outcome of this reaction [8–13] still remains a challenge despite several breakthroughs with homogeneous catalysts [14–18]; one of the most important and difficult targets is the control of the configuration of the double bond, the *E*- and *Z*-selectivity. Most often, high selectivity is only obtained for specific substrates, where thermodynamics favour one isomer, often that with an *E*-configured double bond (styrenyl systems or alkenes with electron withdrawing substituents) [19–21].

Here the discussion will focus on the stereoselectivity of alkene metathesis in order to delineate the current state of the art in the case of heterogeneous catalysts and show how it can be used to characterize active sites as well as to put forward possible strategies to approach the problem.

## Review

### Stereoselectivity in alkene metathesis: a challenge and a tool

**Scheme 1:**
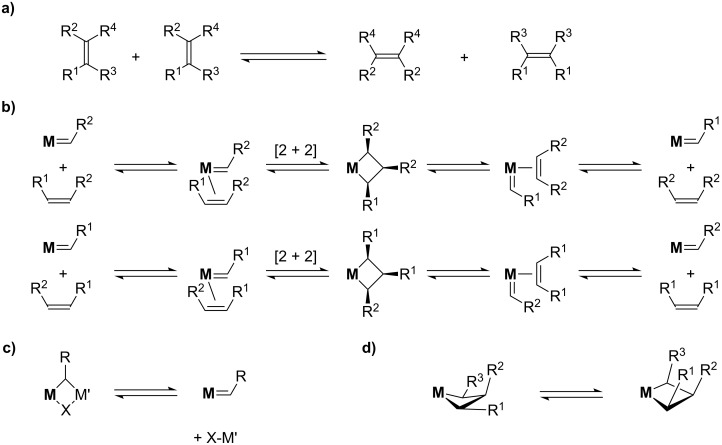
Alkene metathesis mechanism.

Alkene metathesis is a reaction where the alkylidene fragments of alkenes are exchanged (transalkylidenation, Scheme 1a). The mechanism involves at least four steps: alkene coordination, [2 + 2]-cycloaddition generating metallacyclobutanes and the corresponding reverse steps, i.e., cycloreversion and alkene dissociation (Scheme 1b). The approach of an alkene of a given configuration towards a metal–alkylidene intermediate in a given configuration will generate a metallacyclobutane from which a new alkylidene and alkene with specific configurations will be formed. Note that additional steps are possible such as: i) formation of the active alkylidene species or ii) interconversion of metallacyclobutane isomers (TBP vs SBP), however, these typically do not affect the stereochemical outcome of the overall reaction (Scheme 1c and Scheme 1d).

Overall the (*E*/*Z)* ratio of the resulting alkene products can provide information about the whole metathesis process and the structure of the active sites (vide infra) [22–23]. However, because alkene metathesis (for most acyclic alkenes) has a free energy close to 0 and is reversible, the (*E*/*Z*) ratio readily evolves towards a thermodynamic value [(*E*/*Z*) ≥ 3 for di-substituted alkenes] via metathesis, and all the valuable *kinetic stereochemical information* is easily lost, and consequently special care has to be taken in order to obtain useful information from (*E*/*Z*) ratios, i.e., they should be measured at low conversions or contact times.

**Scheme 2:**
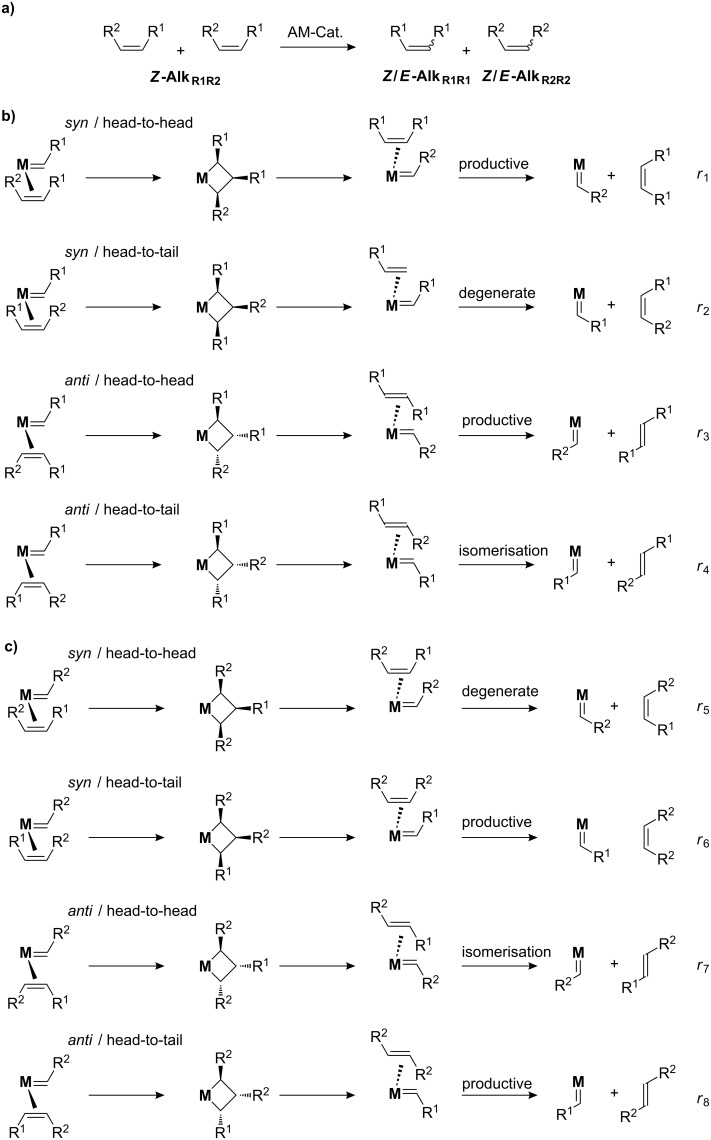
Metathesis possibilities.

As an example, let us analyse the metathesis of a dissymmetric *Z*-alkene, ***Z*-Alk_R1R2_** (R^1^ = R^2^; R^3^ = R^4^ = H), into **Alk_R1R1_** and ***Z*-Alk_R2R2_**. First, such a reaction will lead to the formation of two alkylidene intermediates, M=CHR^1^ and M=CHR^2^, and for each intermediate, the alkene can approach in four possible ways: *syn*/head-to-head, *syn*/head-to-tail, *anti*/head-to-head and *anti*/head-to-tail (Scheme 2).

Of these eight possible pathways, four are productive leading to the (*Z*)- or the (*E*)-alkene products (**Alk_R1R1_** and **Alk_R2R2_**), two are degenerate leaving the reactant untouched (***Z*-Alk_R1R2_** → ***Z*-Alk_R1R2_**), and two yield the alkene reactant with the opposite stereochemistry (***Z*-Alk_R1R2_** → ***E*-Alk_R1R2_**); the latter corresponding to an isomerisation. As the products **Alk_R1R1_** and **Alk_R2R2_** build up in the reaction mixture, they will undergo the same processes, including isomerisation, until the overall thermodynamic equilibrium is reached, typically leading to the formation of the *E*-products for di-substituted alkenes, in particular when one of the substituent is an electron withdrawing group. Any kinetic information will be obtained only at low conversions, where isomerisation is minimal. This can be performed by looking at the (*E*/*Z*) ratio of products at low conversions, but the best approach is to study the evolution of the (*E*/*Z*) ratio of the reactant (*E*/*Z*)_t-reactant_ vs products (*E*/*Z*)_t-product_ as a function of time/conversion and to plot the (*E*/*Z*) ratio of products as a function of the (*E*/*Z*) ratio of the reactants; the latter approach leads to, in most cases, a straight line, any deviation indicating the approach to thermodynamic equilibrium or a change of the active site structure (a full kinetic treatment of this has been provided by Bilhou et al.) [24]*.* The intercept at x = 0 gives the intrinsic stereoselectivity of the catalyst, (*E*/*Z*)_0_, and corresponds to a snapshot of the catalyst at work. From a purely statistical standpoint (Scheme 2), one would expect to observe: i) a one-to-one (*E*/*Z*) kinetic ratio for each alkene products, (*E*/*Z*)_0_ = 1, and ii) the formation of the opposite isomer of the alkene reactant for every two metathesis products transformed. If the catalyst show any selectivity, (*E*/*Z*)_0_ of products will deviate from one. The same analysis can be performed for (*E*)- and terminal (R^2^ = R^3^ = R^4^ = H) alkenes; for the former it is best to study the (*Z*/*E*) ratio rather than the (*E*/*Z*) ratio as a function of time.

Overall, this shows that it is not possible to avoid isomerisation in metathesis and achieving high stereoselectivity is thus difficult, because isomerisation of the starting material will occur as the reaction proceeds at a rate two times lower than metathesis, and self-metathesis of both isomers (of both the starting material and products) will then compete as the product concentration increases. This clearly illustrates the challenge in obtaining high stereoselectivity at high conversions; further underlining the need for highly stereoselective as well as stereospecific catalysts.

Finally, it also shows that monitoring the stereoselectivity at low conversions (*E*/*Z*)_0_ can be very helpful in obtaining molecular information about the structure of the active sites and also how it evolves with time. Stereochemical analysis is therefore a powerful tool that will be exploited thereafter to obtain more information about supported catalysts.

### Stereoselectivity of heterogeneous alkene metathesis catalysts: a snapshot of the structures of active sites

#### Well-defined silica supported catalysts

Metathesis of propene in flow reactors can easily allow the kinetic stereoselectivity of a catalyst at low contact times (high space velocity) to be obtained. For instance, [(≡SiO)(*t-*BuCH_2_)Re(=CH*t-*Bu)(≡C*t-*Bu)] displays a (*E*/*Z*)_0_ of 2, which is very close the thermodynamic equilibrium value of 3, even at low conversions and contact times [25–27]. Switching to Mo- and W-based catalysts that have variety of ligands ([(≡SiO)(X)M(=CHR’)(=NR)], Table 1), in particular with different groups for X and on the imido ligands, the selectivity varies with (*E*/*Z*)_0_ ranging from 1.6 to 0.5. In particular, with the bulky X = NPh_2_ and small imido ligands (*N*-adamantyl), *Z*-selectivity is achieved, albeit never exceeding 67% [(*E*/*Z*) = 0.5]. While low, it shows that it should be possible to control the stereoselectivity by using the right combination of ligands. Note also that these low selectivities are in sharp contrast with the recent results of the groups of Hoveyda and Schrock, who showed that with very bulky aryloxide ligands in place of the siloxy, such systems achieved high levels of stereoselectivity (up to > 95% selectivity at high conversions) [15–18]. This demonstrates that the siloxy ligands on a silica surface should not been viewed as such a large ligand.

**Table 1:** Stereoselectivity of well-defined silica supported catalysts of general formula [(≡SiO)(X)M(=CHR’)(=NR)] with M = Mo or W: (*E*/*Z*)-ratio at low conversions in propene metathesis.

Mo (W)	Reference	Effect of the substituent on the imido ligand (=NR)			
X-ligand		2,6-di-*i-*Pr-C_6_H_3_	Ad	2,6-di-Me-C_6_H_3_	*o*-CF_3_-C_6_H_4_

CH_2_*t-*Bu	[28]	0.8 (1.3) [29]	—	—	—
NPh_2_	[30]	1.5	0.5	0.6	0.8
Pyr	[30]	0.8	0.6	—	—
2,5-di-Me-Pyr	[31]	1.1 (0.8) [32]	1.3	—	0.8
O*t-*Bu	[33]	0.7	—	—	—
OR_F6_	[34]	1.3	—	—	—
OAr	[34]	0.8	—	—	—

Stereoselectivity has been studied in greater details with the Re-based silica supported catalysts [26]. Using ethyl oleate as a representative *Z*-alkene, this catalyst is *Z*-selective with (*E*/*Z*)_0_ values (*Z*-selectivities) ranging between 0.05 (> 95%, diastereoselectivity excess (de) > 90%) and 0.8 (55%, de = 10%), depending on the solvent (THF > toluene > heptane, Table 2); the best compromise between activity and selectivity being achieved in toluene. This *Z*-selectivity can be interpreted as a way to minimize interactions between the surface with all alkyl ligands of the alkene and the alkylidene ligand (Scheme 2).

**Table 2:** Initial rates (TOF) and selectivity at low conversions (*E*/*Z*)_0_ in the metathesis of methyl oleate (0.12 M) with [(≡SiO)(*t-*BuCH_2_)Re(=CH*t-*Bu)(≡C*t*-Bu)] (1 mol %).

Solvent	TOF/min^−1^	(*E*/*Z*)_0_

THF	<0.120	0.05–0.2
Toluene	4.8	0.2–0.4
Heptane	6.6	0.6–0.9

#### Re-based alumina supported systems

With Re-based alumina supported catalysts, the (*E*/*Z*) ratio in 2-butenes in the metathesis of propene is always close to the actual thermodynamic value (ca. 2 vs 3) even for conversions as low as 2% (vs ca. 30% thermodynamic plateau).

**Scheme 3:**
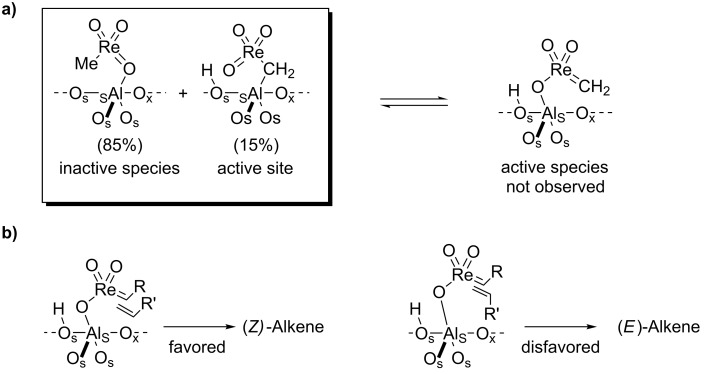
Metathesis with Re-based alumina supported catalysts.

**Figure 1:**
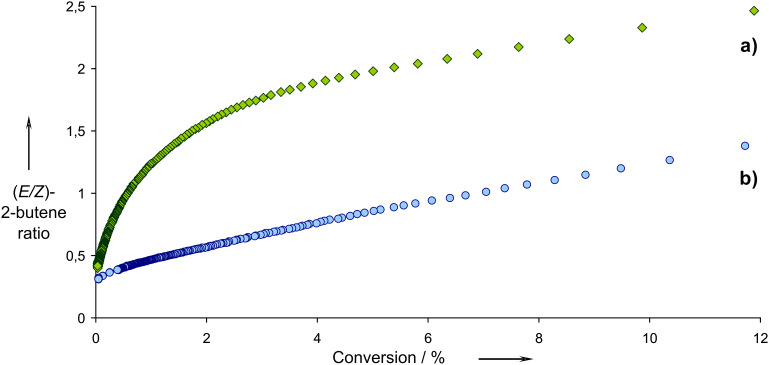
(*E*/*Z*) ratio as a function of conversion. a) MeReO_3_ supported on alumina and b) MeReO_3_ supported on alumina modified by trimethylsilyl functionalities.

For MeReO_3_/Al_2_O_3_, where the active sites is [Al_S_CH_2_ReO_3_] (Scheme 3a) [35–36], decreasing the conversion to well below 0.1% allows the measurement of a kinetic (*E*/*Z*) ratio of 0.4 thus showing that this catalyst is slightly *Z*-selective (70%), probably because the favoured pathway minimizes interaction of the substituents with the surface (Scheme 3b) [36]. Importantly, the evolution of the (*E*/*Z*) ratio in 2-butenes as a function of propene conversion shows hyperbolic behaviour (Figure 1a).

This is reminiscent of Langmuir–Hinshelwood kinetics (or Michaelis–Menten kinetics if it were an enzyme), and in fact, when far from equilibrium, it is possible to express the rate of isomerisation of (*Z*)- into (*E*)-2-butenes via metathesis (*r*_isom_) according to Equation 1, where *k*_isom_ is the rate constant of metathesis and Θ_Z-C4_ the surface coverage in (*Z*)*-*2-butene (*Z-*C4). Surface coverage describes the concentration of a gas at the surface as a function of the partial pressures of all components (*P*_i_) and their equilibrium constant (λ_i_). For (*Z*)-2-butenes, it can be expressed according to Equation 2, which explains the hyperbolic relationship obtained for the evolution of selectivity vs conversion.

[1]
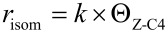


[2]
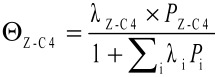


[3]
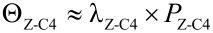


**Scheme 4:**
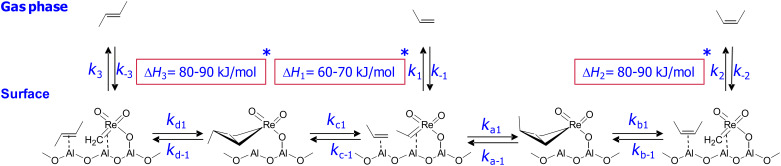
Alkene selectivity of metathesis reactions.

From this equation, it is clear that what drives the selectivity (or the non-selectivity) is that 2-butenes have a better surface coverage than propene (the reactant) because of their lower vapour pressure and higher affinity for the surface (greater Δ*H* of adsorption) [37–38], and therefore reacts faster than propene (Scheme 4). In other words, isomerisation is faster than productive metathesis, so that the thermodynamic ratio of 2-butenes is almost reached even at relatively low conversions. This equation also indicates that modification of adsorption properties of the support should, in principle, modify the selectivity of the catalysts.

The introduction of hydrophobic groups (OSiMe_3_) prior to chemisorption of MeReO_3_ on alumina leads to a completely different behaviour in terms of selectivity (Figure 1b) [39]. Here, the selectivity nearly evolves linearly with respect to conversion, due to a lower surface coverage of alkenes on this modified support (λ_i_*P*_i_ << 1, because of the loss of acidity and, as a result, interaction between alkenes and the alumina surface) so that the rate of isomerisation is now proportional to conversion (Equation 3) allowing higher *Z*-selectivities at higher conversions.

It is also noteworthy that the selectivity at low conversions with this modified catalyst is the same as the previous one, indicating that the structure of the active sites is probably similar in both cases. It is also worth noting that a similar value was also obtained for Re_2_O_7_/Al_2_O_3_ [40–41], which infers similar structural features for the active sites [42].

Finally, this shows that modifying the surface adsorption properties can favour the formation of primary products by slowing down secondary processes such as isomerisation, but that improving the selectivity requires tuning the structure of the active sites to favour one isomer over the other (modification of the first coordination sphere).

#### Hybrid materials containing Ru–NHC units

**Scheme 5:**
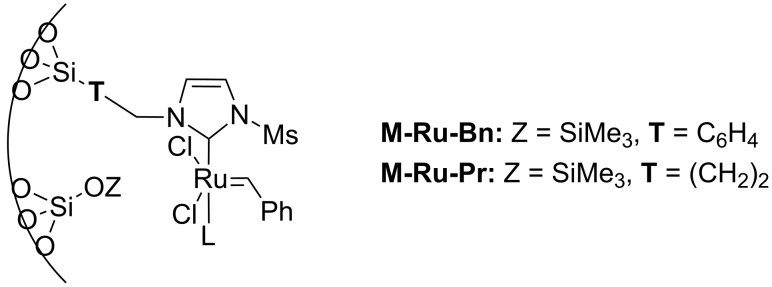
Hybrid organic–inorganic catalyst containing a Ru–NHC unit.

New strategies to develop supported homogeneous catalysts involves the preparation of hybrid organic–inorganic materials [43], where surface functionalities such as typical organic ligands are perfectly distributed within the pore networks of a mesoporous silica. For other approaches used to prepare supported homogeneous catalysts, see the reviews [44–46]. Selective grafting of organometallic complexes onto these pendant ligands can then be performed. Using this technology, several materials containing N-heterocyclic metal units (M–NHC) have been prepared, including a system containing a Ru–NHC unit (Scheme 5), which displayed unprecedented activity in the metathesis of ethyl oleate with turnover numbers in excess of 15,000 [47].

Identity of the active sites for these systems could be obtained from stereochemical studies, i.e., the measurement of (*E*/*Z*)_0_. Indeed, comprehensive studies of various Ru–NHC homogeneous catalysts showed that the so-called G-I catalysts (no NHC and PCy_3_ coordinated to the metal centre) displayed selectivities at low conversions with (*E*/*Z*)_0_ significantly different from G-II catalysts (one NHC ligand): 2.7–3.2 vs 1.5–2.1, respectively (or 3.5–3.6 vs 2.0–2.7, if one looks at (*E*/*Z*)_0_ obtained by extrapolating (*E*/*Z*) ratio to 0 from values obtained under steady state conditions; Table 3). These data show that one can know if active species is based on NHC–Ru as opposed to a Cy_3_P–Ru. Moreover, the change of values between selectivities at low conversions and extrapolated data (values in parentheses) can be interpreted as an indication of a change of structure of active sites during the catalytic run. When applying this study to materials (**M-Ru-Pr** and **M-Ru-Bn**, Scheme 5 and Table 3), a (*E*/*Z*)_0_ of 2.0–2.2 was found irrespective of whether the Ru–NHC containing materials had a propyl or a benzyl pendant group, in clear agreement with the presence of Ru–NHC active sites. This once again demonstrates the power of this method to probe active site structures.

**Table 3:** Stereoselectivity of Ru–NHC catalysts. Comparison of molecular complexes and materials containing Ru–NHC units.

Catalysts	(*E*/*Z*)_0_	
	9-Octadecene	Diester^a^

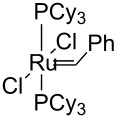	2.7 (3.6)	3.0 (3.4)
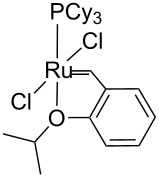	3.2 (3.5)	3.2 (3.5)
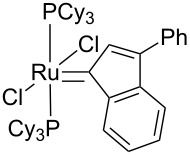	3.2 (3.5)	2.7 (3.4)
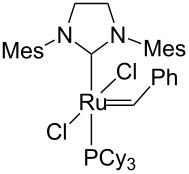	1.5 (2.5)	1.7 (2.7)
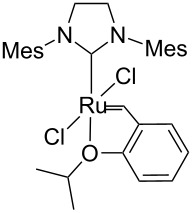	1.6 (2.3)	2.0 (2.5)
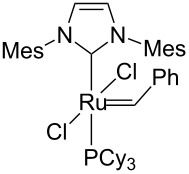	1.7 (2.6)	2.0 (2.6)
**M-Ru-Pr**	—	2.0 (2.0)
**M-Ru-Bn**	—	2.2 (2.2)

^a^The values in parentheses correspond to extrapolated (*E*/*Z*) ratio from the extrapolated value at the steady state.

## Conclusion

Overall, obtaining selectivities at low conversions (*E*/*Z*)_0_ can help to probe the structure of surface species at a molecular level, and should probably be used more often as a probe to understand the structure and the modification of structures of catalysts under working conditions, whether homogeneous or heterogeneous. Ru-based heterogeneous catalysts are *E*-selective (60–70%) when transforming (*Z*)-alkenes such as ethyl oleate, as their homogeneous equivalents. In contrast, the Re-based silica supported catalyst, in a d^0^ configuration, is slightly *Z*-selective (70–95%) under the same reaction conditions.

For the conversion of propene, it is clear that silica supported catalysts are not selective with (*E*/*Z*)_0_ ratio ranging from 0.5 to 2 and that the change of selectivity results from the structure of the ligands (first coordination sphere). Importantly, this low selectivity indicates that a surface siloxy group is not large enough to provide any control of selectivity in contrast to the bulky phenoxy ligands used for highly selective homogeneous catalysts [14–18]. In the case of catalysts supported on alumina, the support plays a major role because it controls the rate of adsorption/desorption of reactants and products. This precludes high selectivity being reached as secondary isomerisation processes via metathesis are favoured.

Overall, the current data show that developing selective heterogeneous catalysts will require developing more tuneable surfaces in particular through the control of the first coordination sphere of the metal centre. This has already been achieved for enantioselective heterogeneous catalysts [48], but remains to be realised for *Z*- or *E*-selective catalysts. Promising routes include the incorporation in a controlled manner of perfectly designed organic functionalities in organic or inorganic matrices [47–48].
